# Editorial: Advanced Imaging Techniques and Imaging Markers of Synaptic Density and Brain Connectivity in Animal Models of Neurological Diseases

**DOI:** 10.3389/fnins.2022.980368

**Published:** 2022-07-19

**Authors:** Anne M. Landau, Nadja Van Camp, Alexandra Petiet

**Affiliations:** ^1^Translational Neuropsychiatry Unit and Department of Nuclear Medicine & PET-Center, Department of Clinical Medicine, Aarhus University, Aarhus, Denmark; ^2^Laboratoire des Maladies Neurodégénératives, Université Paris-Saclay, CEA, CNRS, MIRCen, Fontenay-aux-Roses, France; ^3^Centre de Neuroimagerie de Recherche, Institut du Cerveau, Inserm U1127, CNRS UMR 7225, Sorbonne Universités, Paris, France

**Keywords:** imaging, synaptic density, biomarkers, PET, MRI, neurological diseases

Changes in connectivity, synaptic density or plasticity occur early in the pathology of neurological diseases. Until recently, only a limited number of imaging tools, mostly in the Magnetic Resonance Imaging (MRI) field, could measure changes in connectivity in the living brain. Diffusion-based imaging and tractography have been used to study short and long-range brain anatomical connections while functional imaging of the resting brain has been used to characterize functional connectivity changes. Recently, novel positron emission tomography (PET) imaging ligands based on the structure of levetiracetam have become available which bind specifically to a molecule expressed in presynaptic synapses, synaptic vesicle glycoprotein 2A (SV2A), providing a non-invasive measure of synaptic density for *in vivo* imaging. These are fast emerging research fields, providing new perspectives on innovative biomarkers. This Frontiers Research Topic focused on the application of imaging techniques and markers of connectivity, synaptic density and plasticity in animal models of neurological diseases.

A subset of the articles reviewed aspects of the use of novel PET ligands of synaptic SV2A density. Rossi et al. provided an extensive review of the currently accepted features and functions of the SV2A molecule and an overview of preclinical and clinical studies, shedding light on important questions which remain unanswered such as the true biological roles of SV2A, the entry mechanisms of levetiracetam and SV2A tracers as well as their binding sites, and the varying SV2A expression patterns in different brain structures and disease contexts. Future studies addressing these issues are necessary to determine whether increased tracer binding indicates more SV2A copies, more vesicles or more synapses. A better understanding of the tracer properties and the measurement of synaptic density calls for in depth validation using gold standard molecular biology approaches, reviewed by Serrano et al. Their review provides a critical assessment of challenges and limitations of employing synaptic density biomarkers in the study of brain disorders, and discusses the best implementation of available tools, including measurements in postmortem tissue by electron microscopy, histology, immunohistochemistry and autoradiography and *in vivo* using PET and MRI methods including magnetic resonance spectroscopy and chemical exchange saturation transfer. An important point was the translatability of most of these methods, also used in clinical research. Toyonaga et al. highlight the opportunities of SV2A PET in small animal imaging with a focus on challenges to be overcome with the goal of helping the reader to design and analyse SV2A PET imaging studies in small animals. They present main issues associated with small animal imaging including limited spatial resolution of PET scanners and challenges of kinetic modeling. They compared different injection routes and brain imaging characteristics of currently available SV2A tracers. These complementary articles focus on important aspects of SV2A PET aiming to improve the understanding, the quality of the data, the choice of methods to confirm *in vivo* findings and the best use of small animal models in hopes of raising the impact of this non-invasive tool in neuroscience research and drug development.

A major question among PET researchers is the nature of the relationship between the novel SV2A ligands and FDG, the most commonly used PET ligand, which measures glucose metabolism. Raval et al. imaged 6-hydroxydopamine (6-OHDA) lesioned rodents with unilateral dopamine depletion, with 11C-UCB-J and 18F-FDG, in one of the first studies to directly compare synaptic SV2A density with glucose metabolism. As expected, there was a correlation between the two tracers, both showing decreases in the ipsilateral striatum of 6-OHDA injected rats. However, their exploratory studies in extrastriatal regions implied a discordance in the direction of change in binding between the two tracers, and this interesting finding should be explored in further studies.

A variety of techniques other than the classical diffusion and functional MRI methods are being developed to study brain connectivity and are the topic of much ongoing research, some of which are presented in this Topic. Functional ultrasound can be used to assess cerebral brain volume changes associated with resting-state functional connectivity modulations in animal models of neurological disorders. Droguerre et al. used this technique in a hypertensive rat model and revealed altered local and long-range functional connectivity consistent with clinical findings. This study illustrates the potential for functional ultrasound as a translational tool in Attention Deficit Hyperactivity Disorder research.

Repetitive Transcranial Magnetic Stimulation (rTMS) has been used to treat symptoms of neurological and psychiatric disorders, but its underlying mechanisms are poorly known. Arceves-Serrano et al. reviewed how PET and MRI can play a role in a better understanding of the physiology, morphology, and neuropharmacology effects of rTMS, including the effects on neurotransmission, brain activity, resting-state connectivity and microstructure. They highlight that these are complex and multifactorial, and depend on technical settings, but also on clinical states and patient populations. This knowledge should help clinicians select optimal protocols to stratify patient populations.

Measures of altered cognition of complex tasks such as navigation and orientation occur early in Alzheimer's disease (AD). Badea et al. proposed a novel metric, the absolute winding number, to characterize early changes in AD mouse models carrying different APOE gene mutations. The authors evaluated spatial search strategies through the shape of the swim path of the animals. This approach has the potential to identify altered circuits associated with cognitive changes in prodromal stages of AD.

Neuronal tracing is a classic and well-established method to study neuronal connectivity. A study by Steinmuller et al. contributes new insight into the neuroanatomical features and connectivity of the minipig basal ganglia, demonstrating the existence of a hyperdirect pathway from cortical areas to the subthalamic nucleus and outlining cortico-striatal motor connections. The minipig is being increasingly used as a non-primate translational large animal model in neuroscience studies, and these findings are of particular relevance to studies of Parkinson's disease and deep brain stimulation.

Overall, the search for new imaging techniques and biomarkers of structural and functional connectivity changes in neurological disorders, and the use of novel PET ligands to assess synaptic SV2A density, should help to improve our understanding of pathophysiological states and provide new translational tools for clinical use.

## Author contributions

All authors listed have made a substantial, direct, and intellectual contribution to the work and approved it for publication.

## Conflict of interest

The authors declare that the work was conducted in the absence of any commercial or financial relationships that could be construed as a potential conflict of interest.

## Publisher's note

All claims expressed in this article are solely those of the authors and do not necessarily represent those of their affiliated organizations, or those of the publisher, the editors and the reviewers. Any product that may be evaluated in this article, or claim that may be made by its manufacturer, is not guaranteed or endorsed by the publisher.

